# Hyoscyamia

**Published:** 1881-02

**Authors:** 


HYOSCYAMIA, a crystalline alkaloid, is odorless, bitter, and quite insoluble in cold water; it is easily soluble in hot water, alcohol, ether and chloroform. The best preparation is that of Merck, and it is efficient in the dose of one-thirtieth of a grain; one quarter of a grain could not be given without great danger. It possesses the hypnotic, anodyne, and antispasmodic properties of hyoscyamus, and from its small bulk may be given in coffee, etc.—a great convenience in cases of mania, where patients refuse medicine. In the author's opinion it is especially efficacious in the treatment of mania, delirium tremens, and paralysis agitans; and is particularly to be preferred when the depressing effects of the bromides upon the heart and respiration are feared. Short histories are given of three cases of puerperal mania and one of delirium tremens successfully treated with hyoscyamia; also of one case of paralysis agitans where its administration was attended with benefit. The dose of one-thirtieth of a grain was repeated every hour or two until slight mydriasis and dryness of the mouth were produced.

